# Investigating the efficiency of medical and health resources and its influencing factors in the Yangtze River Delta urban agglomerations: based on the undesirable super-efficiency SBM-Malmquist-Tobit model

**DOI:** 10.3389/fpubh.2025.1527424

**Published:** 2025-05-09

**Authors:** Yi Chen, Qianlong Li, Yuting Zhang, Xiaoya Zhang, Handong Zheng

**Affiliations:** ^1^School of Business, Anhui University, Hefei, China; ^2^School of Management, Hefei University of Technology, Hefei, China

**Keywords:** efficiency of medical and health resources, influencing factors, the Yangtze River Delta urban agglomerations, undesirable super-efficiency SBM model, Malmquist index, Tobit regression model

## Abstract

The medical and health resources in the Yangtze River Delta region are abundant, occupying a significant position in China’s medical and health system with wide service coverage. The efficient allocation of medical and health resources in this region is a key issue affecting the high-quality development of medical and health services. Existing research on the representative region of the Yangtze River Delta Urban Agglomeration remains relatively limited, with insufficient analysis of the efficiency of medical and health resource allocation in the area, as well as a lack of in-depth exploration into its influencing factors and barriers. This study employs the undesirable output super-efficiency SBM model to calculate the static super-efficiency values of medical and health resource allocation in 41 cities of the Yangtze River Delta urban agglomeration from 2015 to 2020. The Malmquist index is utilized to analyze the changes in dynamic efficiency, and the panel Tobit regression model is utilized to analyze the influencing factors of comprehensive efficiency of medical and health resource allocation in the Yangtze River Delta urban agglomeration. The study highlights that the Yangtze River Delta urban agglomeration confronts multiple systemic challenges, such as pronounced disparities in healthcare resource allocation, uneven dissemination of medical technological innovations, insufficiently effective policy implementation, severe pandemic-induced impacts, enduring gaps in urban–rural healthcare resources, and financial accessibility barriers in low-efficiency cities. By integrating empirical evidence from existing studies and performing comparative case analyses, this research puts forward actionable recommendations. These strategies are designed to improve the equity and efficiency of medical and health resource allocation within the region, while offering transferable insights for other regions facing similar institutional and operational challenges.

## 1 Introduction

The uneven distribution of medical and health resources represents a significant global challenge, and promoting their effective allocation has become an issue of worldwide concern (1). As the most populous country in the world, China’s efficiency in allocating medical and health resources will have a profoundly important impact on public well-being and social development. The effective implementation and comprehensive advancement of the Healthy China strategy have driven substantial progress in China’s healthcare sector. However, structural issues remain prevalent in China’s medical and health service system. Existing research has identified that the imbalance in the allocation of medical and health resources in China constitutes one of the most severe and pressing public service challenges (2).

The Yangtze River Delta (YRD) region is one of China’s most dynamic and densely populated areas, characterized by early regional integration. Despite occupying less than 4% of China’s land area, it generates approximately one-quarter of the country’s total economic output. However, the rapid development of the YRD urban agglomeration has also intensified intra-regional disparities and increasingly uneven development among cities. These disparities stem from significant variations in initial policy support, geographic location, economic level, and infrastructure across different cities. Notably, in the healthcare sector, the imbalance within the YRD urban agglomeration has become particularly pronounced (3). In the face of structural problems in the allocation of medical and health resources, the YRD urban agglomeration has implemented numerous healthcare reform policies and measures. However, the outcomes of these initiatives remain insufficiently evident. The structural imbalance in healthcare resource allocation continues to pose a significant obstacle to the development of healthcare in the YRD urban agglomeration. Against this backdrop, this study selects the YRD region as the research object to analyze the current status and dynamic changes in the distribution of medical and health resources within the YRD city agglomeration. Furthermore, it investigates the influencing factors behind these disparities. Based on these findings, the study aims to provide more targeted recommendations for the YRD urban agglomeration by drawing on existing case studies. This will facilitate a more effective resolution of the issue of uneven medical resource distribution in the YRD and promote the high-quality and integrated development of medical and healthcare services in the region.

With the development of the healthcare industry and the deepening of medical and health system reforms in China, scholars have examined the efficiency of China’s medical and health service system from multiple perspectives, including government investment, hospital operations, health services, and medical care. From a research standpoint, these studies primarily fall into two categories. On the one hand, from a national-level perspective, Shen and Sun (2) demonstrated that government subsidies enhance regional medical quality and hospital efficiency by analyzing healthcare efficiency metrics across 31 provinces in China. Zhou et al. (4) identified substantial disparities in both the level and capacity of medical services among Chinese provinces, with higher medical efficiency predominantly observed in the developed eastern coastal regions. On the other hand, from a regional-level perspective, Zhou et al. (4) revealed through panel data analysis in Jiangsu Province that the productivity of community health service centers has markedly increased, driven primarily by technological progress. Cheng et al. (5) conducted a study with county-level hospitals in Henan Province as samples, and found that the technical efficiency of these hospitals was greatly improved by strict bed capacity management, reducing the average length of stay, optimizing the ratio of medical staff to beds, and other measures. From a methodological perspective, Data Envelopment Analysis (DEA) has been widely used in the study of resource allocation efficiency due to its ability to effectively address efficiency issues under complex backgrounds of multiple inputs and outputs. By generating weight values through mathematical programming to enhance the objectivity of decision unit evaluation, DEA has gradually been combined with other analytical methods in practice to compensate for its shortcomings. For example, in the research conducted by Du (6), DEA and Tobit models were used to measure the efficiency values and influencing factors in the eastern, central, and western regions of China. Liu et al. (7) measured the efficiency of pediatric public services in China using a three-stage SBM-DEA model.

Meanwhile, a limited number of scholars have also conducted studies on the healthcare development of the YRD urban agglomeration. For public health conditions, Li et al. (8) revealed that downstream emissions from the Yangtze River could negatively impact public health in the YRD region. Sun et al. (9) demonstrated that haze pollution adversely affects public health levels, while urbanization rates and the number of health technicians significantly enhance public health efficiency. Li et al. (10) highlighted the pronounced spatial differentiation of public service levels in the YRD, with a decreasing gradient from east to west. The influencing factors driving this spatial–temporal evolution include urbanization levels, economic development, industrial structure, and regional population size. To address healthcare resource allocation, Shen and Sun (2) employed the entropy weight method and other approaches to analyze the spatial correlation and convergence of medical and health resources in the YRD urban agglomeration, revealing significant spatial correlations and clustering effects among provinces and cities. Li et al. (11), through an investigation of the spatio-temporal evolution and heterogeneous impacts of healthcare service supply levels in the YRD, found that population density and urban–rural income disparities exert negative spatio-temporal influences on healthcare service supply levels, whereas per capita GDP at the urbanization level has a positive spatio-temporal effect. Jing et al. (3) utilized an anisotropic growth model to elucidate the dynamic interactions between the number of hospital beds, physicians, and urban economic indicators in the YRD urban agglomeration, emphasizing the critical role of economic factors in shaping healthcare resource distribution.

The limitations of the existing studies are summarized as follows: Firstly, there is a relative scarcity of research on the efficiency of medical and health resource allocation in the YRD Urban Agglomeration, which serves as a representative region. Secondly, the methodologies employed in current studies on medical and health resource allocation in this urban agglomeration tend to be relatively monotonous, lacking integration of multiple approaches for comprehensive measurement and evaluation of both the regional issues and potential solutions. Additionally, the analysis of influencing factors in existing research remains overly simplistic, focusing narrowly on specific perspectives without adequately considering the significant impacts of socio-economic environments, financial support, and healthcare systems on regional healthcare resource efficiency. Furthermore, empirical analyses in existing studies predominantly remain at the level of descriptive data, lacking case comparisons and in-depth exploration of the underlying problems. To address these gaps and deficiencies, this study focuses on the efficiency of medical and health resource allocation in the YRD urban agglomeration. It employs the undesirable super-efficiency SBM model to calculate static health resource allocation efficiency in the region from 2015 to 2020, applies the Malmquist index to measure and analyze the dynamic efficiency of medical and health resources across different cities, and utilizes Tobit regression analysis to identify influencing factors under varying efficiency levels. Determine the influencing factors at different efficiency levels and explore the optimization path of medical and health resource allocation. The marginal contributions of this study are as follows: first, selecting the YRD urban agglomeration as a focal region for analysis enriches the existing research findings on this area. Second, by adopting the undesirable super-efficiency SBM-Malmquist-Tobit model, this study enhances the research methodology, enabling comprehensive measurement of both static and dynamic efficiencies in the region, as well as the underlying influencing factors. Additionally, this study integrates multiple dimensions to analyze the variability in the degree of influence exerted by economic, social, financial, and health system resource conditions on the efficiency of medical and health resource allocation within the YRD, thereby providing a more holistic and systematic understanding of the issue. Finally, through the integration of existing research findings and case-based comparative analysis, this study offers practical recommendations for addressing the problems revealed by empirical analysis, contributing to narrowing regional disparities in healthcare service levels within the YRD and promoting the equalization of healthcare services. Furthermore, this study serves as a valuable reference for future research and other regions aiming to achieve high-quality and coordinated healthcare development.

## 2 Methods

### 2.1 Indicators selection

#### 2.1.1 Efficiency measurement indicators

This study focuses on the evaluation of medical and health resource allocation efficiency in the YRD urban agglomeration. Drawing upon the relevance of existing literature (12, 13) and adhering to the principle of data availability, we establish an indicator system while considering the influence of regional population differences. From the perspectives of healthcare manpower input and healthcare physical input, we select the number of healthcare institutions per city, the number of hospital beds per thousand population (12), and the number of healthcare technical personnel per thousand population (4) as input indicators. Integrating social and economic benefits, we choose the number of outpatient and emergency visits per city (14) and the bed utilization rate (15) as output indicators. Additionally, recognizing the impact of undesirable outputs on social benefits, we select the proportion of medical expenses to total household consumption expenditure (16) as the undesirable output indicator. The efficiency measurement indicator system is illustrated in Table 1.

**Table 1:** Undesirable super-efficiency SBM input–output indicator system.

Dimension	Indicators	Description
Inputs	Number of healthcare institutions per city	The total number of hospitals, primary healthcare institutions, specialized public health institutions, and other healthcare institutions
	Number of hospital beds per thousand population	The ratio of the number of beds in healthcare institutions to the total resident population in each city (unit: per thousand people)
	Number of healthcare technical personnel per thousand population	The ratio of the number of health technical personnel to the total resident population in each city (unit: per thousand people)
Outputs	Number of outpatient and emergency visits per city	Total outpatient and emergency visits at various medical and health institutions
	Bed utilization rate	The ratio of total bed-days used by medical and health institutions in each city to the actual number of days open
Undesirable outputs	The proportion of medical expenses to total household consumption expenditure	Percentage of total consumer expenditure spent on medical expenses by all residents in each city

#### 2.1.2 Influencing factors indicators

The operational efficiency of the medical and health system is simultaneously influenced by various factors, both internal and external. Existing research has explored the impact of external factors such as economic, social policies and other macro and micro-level factors on efficiency. It has been found that factors such as per capita GDP, urban–rural income disparity (17, 18), population density, aging dependency ratio, education level (4), hospital scale, inter-hospital trust relationships (19, 20), fiscal decentralization degree, and segmentation of medical insurance systems (21) have varying degrees of impact on the efficiency of medical and health resource allocation. Furthermore, current studies have analyzed internal factors affecting efficiency within the healthcare system, revealing that factors such as doctor-to-nurse ratio, bed turnover rate, and outpatient and inpatient volumes can influence the efficiency of medical and health resource allocation (22). To comprehensively investigate the influencing factors affecting the efficiency of medical and health resource allocation in the YRD urban agglomeration, this study selects these important factors detailed in Table 2.

**Table 2:** Tobit regression impact factor indicator system.

Perspective	Influencing factors	Description
Economic and social environment	Urbanization rate	Urban population total/Total population of each city *100%
	Per capita GDP	GDP total/Total population
	Urban–rural income gap index	Per capita disposable income of urban residents/Per capita disposable income of rural residents
Financial support level	Proportion of healthcare fiscal expenditure	Medical and health expenditure/general public budget expenditure *100%
	Fiscal autonomy	General public budget revenue/general public budget expenditure *100%
Internal healthcare system	Doctor-to-nurse ratio	Total number of licensed physicians/total number of registered nurses *100%
	The ratio of medical and nursing staff to health technical personnel	The sum of the total number of licensed physicians and the total number of registered nurses/total number of healthcare technicians. *100%
	The average length of hospital stay for discharged patients	Total inpatient days for each medical institution/total number of discharged patients from each medical institution

### 2.2 Data sources

This study focuses on 41 cities in the YRD region. Adhering to the principles of authority, objectivity, and accessibility, the data for this study are sourced from the Statistical Yearbooks of Anhui Province (2016–2021), Zhejiang Province (2016–2021), Jiangsu Province (2016–2021), Shanghai Municipality (2016–2021), Jiangsu Provincial Health Statistics Yearbook (2016–2021), as well as data obtained through information disclosure requests to various provincial and municipal governments.

### 2.3 Model selection

#### 2.3.1 Undesirable super-efficiency SBM model

The DEA method is a non-parametric technique for relative efficiency analysis based on the comparative evaluation of objects. The super-efficiency SBM model overcomes the limitation of the DEA model’s maximum value being restricted to 1, allowing for a quantitative comparison of values on the frontier. Therefore, this study selects the super-efficiency SBM model as the efficiency measurement model. Building upon the super-efficiency SBM model, this study considers undesirable outputs, leading to a non-angle-based super-efficiency SBM model that incorporates consideration of undesirable outputs (23). The expression is as follows:


(1) $$\begin{array}{l} \sigma^{*}=\min_{\lambda ,s^{-},s^{+}}\frac{1+\frac{1}{m}\sum_{i=1}^{m}\frac{s_{i}^{-}}{x_{\mathrm{io}}^{t}}}{1-\frac{1}{q+h}(\sum_{r=1}^{q}\frac{s_{r}^{+}}{y_{\mathrm{ro}}^{t}}+\sum_{k=1}^{h}\frac{s_{k}^{-}}{b_{\mathrm{ko}}^{t}})}\\ \;s.t.\;x_{\mathrm{io}}^{t}\geq \sum_{t=1}^{T}\sum_{j=1,j\neq o}^{n}\lambda_{j}^{t}x_{\mathrm{ij}}^{t}-s_{i}^{-}\;i=1,2,\ldots ,m;\\ \;y_{\mathrm{ro}}^{t}\leq \sum_{t=1}^{T}\sum_{j=1,j\neq o}^{n}\lambda_{j}^{t}y_{\mathrm{rj}}^{t}+s_{r}^{+}\;r=1,2,\ldots ,q;\\ \;b_{\mathrm{ko}}^{t}\geq \sum_{t=1}^{T}\sum_{j=1,j\neq o}^{n}\lambda_{j}^{t}b_{\mathrm{kj}}^{t}-s_{k}^{-}\;k=1,2,\ldots ,h;\\ \;\lambda_{j}^{t}\geq 0(\forall j),s_{i}^{-}\geq 0(\forall i),s_{r}^{+}\geq 0(\forall r),s_{k}^{-}\geq 0(\forall k) \end{array}$$

The objective function of this model, $\sigma^{*}$ represents the optimal solution, which is the super-efficiency value measured by the super-efficiency SBM model; *i* represents the number of input variables, ranging from 1 to *m*; *r* represents the number of output variables, ranging from 1 to *q*; *k* represents the number of non-expected output variables, ranging from 1 to *h*; $s_{i}^{-}$ denotes the slack variables for inputs, $s_{r}^{+}$ denotes the slack variables for outputs, and $s_{k}^{-}$ denotes the slack variables for non-expected outputs, all of which are greater than 0; $\lambda^{t}$ represents the weights.

#### 2.3.2 Malmquist index

Although the undesirable output super-efficiency SBM model can systematically and comprehensively reflect the differences in the efficiency of medical and health resource allocation within the YRD urban agglomeration, further analysis of the dynamic characteristics of medical and health resource efficiency across provinces and cities and the underlying reasons, requires the introduction of the Malmquist index. The Malmquist index, proposed by Swedish economist Malmquist (24) in 1953, addresses the limitations of static analysis. It represents the change in productivity of decision-making units from period *t* to *t + 1*. Further extended by scholars such as Färe and Grosskopf (25), the Malmquist index is decomposed into changes in technical efficiency and technological progress, with the technical efficiency index further decomposable into pure technical efficiency and scale efficiency indexes. It is applied widely in measuring total factor productivity changes in production activities. The formula for calculating the Malmquist index is as follows:


(2) $$\begin{array}{c} \begin{array}{l} M_{i}(x^{t+1},y^{t+1},x^{t},y^{t})\\ ={\left[(\frac{D^{t}(x^{t+1},y^{t+1})}{D^{t}(x^{t},y^{t})})(\frac{D^{t+1}(x^{t+1},y^{t+1})}{D^{t+1}(x^{t},y^{t})})\right]}^{\frac{1}{2}} \end{array}\\ \begin{array}{l} =\frac{D^{t+1}(x^{t+1},y^{t+1})}{D^{t}(x^{t},y^{t})}\times \\ {\left[(\frac{D^{t}(x^{t+1},y^{t+1})}{D^{t+1}(x^{t+1},y^{t+1})})(\frac{D^{t}(x^{t},y^{t})}{D^{t+1}(x^{t},y^{t})})\right]}^{\frac{1}{2}} \end{array} \end{array}$$

In Equation 2, when $M_{i}$ > 1, it indicates an increase in total factor productivity compared to the previous period. If not, it denotes a decrease. Here, $x^{t},y^{t}$ represent the input and output values at time *t*, respectively, and $D^{t}$ is the distance function of the evaluation object with time *t* as the technical reference. This study employs the Malmquist index to analyze the input–output dynamic efficiency of medical and health resources in the YRD urban agglomeration in China from 2015 to 2020, aiming to explore the impact of differentiated inputs such as infrastructure and human resources on the dynamic efficiency changes in medical and health resource allocation.

#### 2.3.3 Tobit model

Due to the non-negative nature of efficiency values measured by the undesirable output super-efficiency SBM model, the use of the Ordinary Least Squares (OLS) method can lead to biased estimation results. Therefore, the Tobit model can be employed for analysis (26) to effectively address issues such as parameter bias. In this study, the comprehensive efficiency of medical and health resource allocation in the YRD urban agglomeration of China is taken as the dependent variable. Representative indicators from different levels are selected as independent variables to conduct a regression analysis on factors influencing healthcare resource allocation efficiency. Considering the panel data utilized in this study, to more fully leverage the information contained within the data, minimize standard errors, and enhance estimation efficiency, this study employs a panel Tobit model for regression analysis. Consequently, the influencing factors of medical resource allocation efficiency in the YRD urban agglomeration can be identified and assessed with greater precision.

## 3 Results

### 3.1 Static efficiency measurement based on undesirable super-efficiency SBM model

This study employs the iDEA Ultra software to evaluate the efficiency of medical and health resource allocation in the YRD urban agglomeration in China from 2015 to 2020 using the undesirable super-efficiency SBM model. It analyzes the efficiency levels of various provinces and cities within the region and performs a comparative assessment. The efficiency calculation results are displayed in Tables 3, 4.

**Table 3:** The comparison of average efficiency in medical and health resource allocation between the three provinces and one city in the Yangtze River Delta in 2015 and 2020.

Region	2015			2020		
	crste	vrste	scale	crste	vrste	scale
Anhui	0.520	0.622	0.897	0.747	0.840	0.890
Jiangsu	0.580	0.705	0.856	0.754	0.790	0.949
Zhejiang	0.576	0.605	0.955	0.866	0.888	0.975
Shanghai	1.277	1.315	0.971	1.218	1.266	0.962
The Yangtze River Delta	0.572	0.661	0.901	0.793	0.848	0.933

crste represents overall efficiency, vrste represents pure technical efficiency, scale represents scale efficiency. The crste, vrste and scale are all derived from the average of all cities in the province.

**Table 4:** The comprehensive efficiency values of medical and health resource allocation in 41 cities in the Yangtze River Delta urban agglomeration from 2015 to 2020.

Region	2015	2016	2017	2018	2019	2020	Average	Rank
Hefei	0.559	0.820	0.817	0.832	0.725	1.022	0.796	16
Huaibei	0.444	0.671	0.612	1.015	0.730	0.719	0.699	19
Bozhou	1.094	1.090	1.060	1.017	1.028	0.727	1.003	4
Suzhou	0.742	0.737	0.874	1.026	1.035	1.009	0.904	9
Bengbu	0.420	0.553	0.518	0.654	1.008	0.727	0.647	21
Fuyang	0.427	0.608	0.550	0.497	0.623	0.695	0.567	29
Huainan	0.428	0.468	0.485	0.586	0.595	0.505	0.511	36
Chuzhou	0.517	0.658	0.591	0.760	0.718	0.578	0.637	22
Lu’an	0.394	1.008	1.009	1.045	1.038	1.032	0.921	8
Maanshan	0.409	1.022	0.865	1.011	1.006	1.047	0.893	10
Wuhu	0.414	0.529	0.500	0.587	0.770	0.598	0.566	31
Xuancheng	0.359	0.607	0.501	0.599	0.691	0.683	0.573	28
Tongling	1.242	1.095	1.114	1.063	1.085	1.058	1.109	2
Chizhou	0.276	0.410	0.339	0.458	0.435	0.440	0.393	40
Anqing	0.370	0.610	1.020	0.695	0.788	0.653	0.689	20
Huangshan	0.226	0.306	0.303	0.376	0.377	0.462	0.342	41
Nanjing	0.669	0.819	0.785	0.760	0.819	0.757	0.768	17
Wuxi	0.579	0.657	0.607	0.602	0.629	0.669	0.624	24
Xuzhou	0.527	0.515	0.521	0.507	0.538	0.539	0.525	34
Changzhou	0.689	0.965	0.752	0.827	0.870	0.919	0.837	14
Suzhou	1.007	1.043	1.015	1.022	1.012	1.007	1.018	3
Nantong	0.510	0.570	0.506	0.530	0.663	0.727	0.584	27
Lianyungang	0.412	0.423	0.422	0.416	0.445	0.533	0.442	39
Huai’an	0.535	0.523	0.518	0.555	0.527	0.681	0.557	32
Yancheng	0.471	0.656	0.507	0.575	0.599	0.591	0.566	30
Yangzhou	0.556	0.847	0.672	0.881	1.008	1.040	0.834	15
Zhenjiang	0.655	1.010	1.005	1.069	1.060	1.020	0.970	6
Taizhou	0.480	0.561	0.501	0.570	0.530	0.659	0.550	33
Suqian	0.444	0.485	0.483	0.529	0.527	0.658	0.521	35
Hangzhou	0.532	0.614	0.552	0.606	0.613	0.643	0.593	26
Ningbo	0.661	1.020	0.924	1.015	1.139	1.004	0.961	7
Wenzhou	1.019	1.022	1.034	1.035	0.871	1.000	0.997	5
Jiaxing	0.650	1.004	0.818	0.911	0.927	1.008	0.886	12
Huzhou	0.539	1.009	1.015	0.690	0.763	1.009	0.838	13
Shaoxing	0.518	0.566	0.533	0.661	0.687	0.645	0.602	25
Jinhua	0.475	0.483	0.485	0.626	0.706	1.004	0.630	23
Quzhou	0.598	0.406	0.375	0.409	0.445	0.529	0.460	37
Zhoushan	0.495	1.017	0.730	1.025	1.010	1.054	0.889	11
Taizhou	0.531	0.668	0.643	0.808	0.913	1.012	0.762	18
Lishui	0.317	0.376	0.379	0.485	0.529	0.618	0.451	38
Shanghai	1.277	1.274	1.271	1.220	1.237	1.218	1.249	1
Average	0.572	0.725	0.688	0.745	0.774	0.793	0.716	—

**Figure 1:**
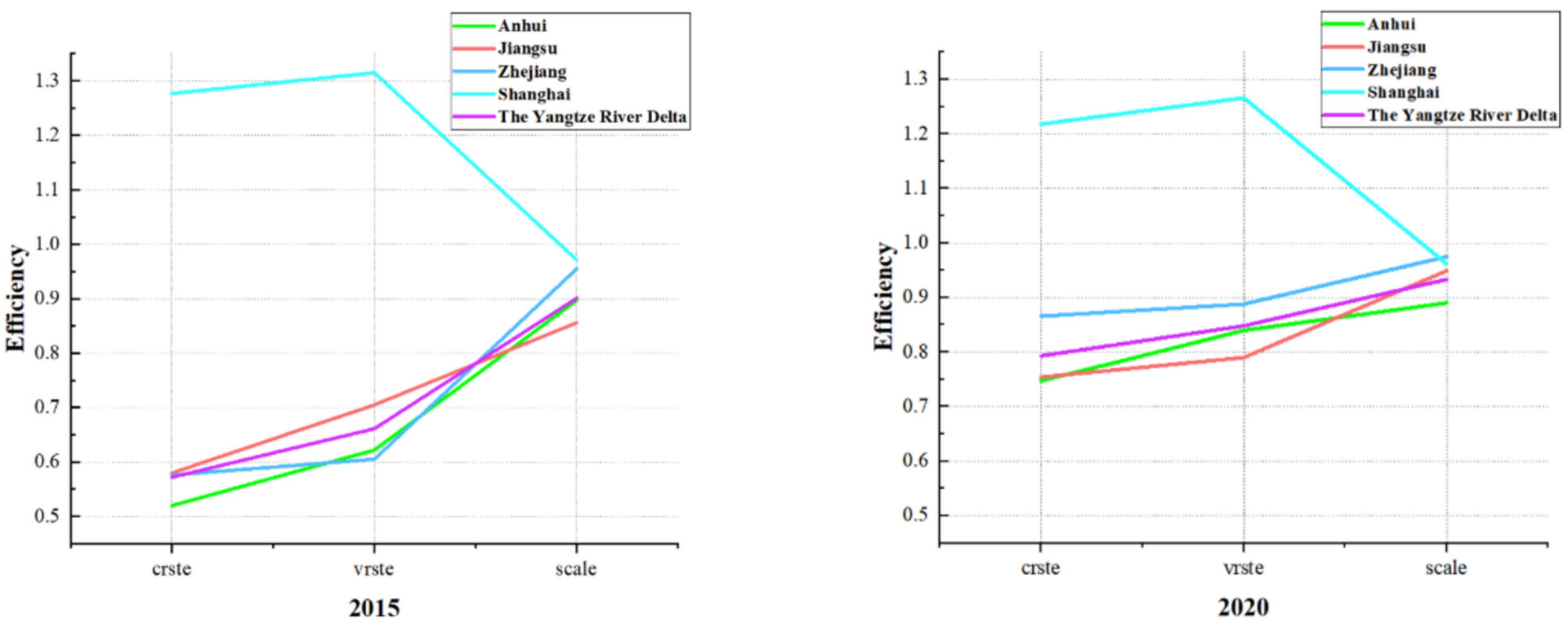
The comparison of average efficiency in medical and health resource allocation between the three provinces and one city in the YRD in 2015 and 2020.

According to Table 3, at the overall level of the YRD region, the average comprehensive efficiency of medical and health resource allocation was 0.793 in 2020. Except for Shanghai, which witnessed a slight decrease compared to 2015, the other three provinces experienced significant increases. The regional efficiency trend shows Shanghai > Zhejiang > Jiangsu > Anhui. Furthermore, as illustrated in Figure 1, it is visually evident that the efficiency gaps between Zhejiang, Jiangsu, and Anhui provinces and Shanghai have been continuously narrowing over the 6 years from 2015 to 2020. This indicates that these three provinces have been consistently optimizing their medical and health resource allocation and achieving notable results. Analysis of efficiency decomposition reveals that changes in pure technical efficiency have played a predominant role in the efficiency variations of the three provinces and one municipality in the YRD region compared to 2015. This suggests that they have been continuously enhancing their capabilities in managing medical and health resources, thereby improving the level and efficiency of resource utilization. Meanwhile, the gap between Anhui Province and Jiangsu Province has gradually narrowed, largely due to a 35.05% increase in pure technical efficiency in Anhui Province. However, Anhui Province has not surpassed Jiangsu Province primarily because of the widening gap in scale efficiency, with Jiangsu Province increasing by 10.86% while Anhui Province experiencing a slight decrease. Therefore, this implies that Anhui Province needs to continue increasing investment and coordination in medical and health resources to improve scale efficiency and approach the optimal input–output scale. Additionally, Jiangsu Province should enhance its resource management capabilities and resource utilization efficiency to better leverage its pure technical efficiency inputs.

Table 4 displays the comprehensive efficiency values of medical and health resource allocation in 41 cities within the YRD urban agglomeration. The analysis indicates substantial disparities in these efficiency values among the cities. Longitudinally, most cities demonstrate an upward trend in their comprehensive efficiency values. However, the degree of change in these values from 2015 to 2020 varies significantly across different prefecture-level cities, with notable fluctuations observed. This suggests that the allocation and management of medical and health resources in most cities are improving, with yearly enhancements in efficiency. Nevertheless, it also underscores significant disparities in resource allocation among cities within the YRD region, reflecting uneven distribution and considerable gaps in both resource allocation and management capabilities across regions. Moreover, this implies variations in resource input and management capacities across areas, causing some cities to struggle in maintaining a stable upward trajectory. Horizontally, the disparity in the efficiency of medical and health resource allocation among the 41 cities in the YRD region remains relatively stable, with no significant short-term fluctuations in resource allocation efficiency. Therefore, each city should prioritize long-term strategic planning to enhance efficiency. Based on the average values from 2015 to 2020, four cities have comprehensive efficiency values exceeding 1, ranked in descending order: Shanghai, Tongling, Suzhou, and Bozhou. Despite differences in economic development levels and medical investment capabilities, these cities all emphasize the development of medical infrastructure, continuously promote the rational use of resources, implement a series of healthcare management policies, and attract a large number of medical professionals, thereby improving their medical resource management capabilities and resource allocation efficiency. On the other hand, the higher medical resource allocation efficiency in cities such as Bozhou and Tongling is closely linked to Bozhou’s focus on medical development as the “Capital of Chinese Medicine” and Tongling’s vigorous implementation of comprehensive medical reforms centered on the construction of tightly integrated urban–rural medical consortia. These measures enable both cities to achieve better medical resource management and higher overall medical resource allocation efficiency. In contrast, there are four cities with efficiency values below 0.5, namely Huangshan, Chizhou, Lianyungang, Lishui, and Quzhou. These cities still face challenges in areas such as bed utilization and the effective use of medical institutions, with many healthcare resources remaining underutilized, indicating substantial potential for improvement in medical and health resource allocation efficiency.

### 3.2 Dynamic efficiency measurement based on the Malmquist index

This study further evaluates the dynamic efficiency of medical and health resource allocation across provinces and cities from 2015 to 2020 by employing the Malmquist index. The results, obtained through the iDEA Ultra software, are summarized in Tables 5, 6.

**Table 5:** The mean Malmquist index and its decomposition index of the three provinces and one city in the Yangtze River Delta from 2015 to 2020.

Province	Effch	Techch	Pech	Sech	Malmquist index
Anhui	1.125	0.815	1.126	1.011	0.890
Jiangsu	1.064	0.867	1.050	1.036	0.908
Zhejiang	1.110	0.868	1.103	1.008	0.941
Shanghai	0.991	0.962	0.993	0.998	0.954
The Yangtze River Delta	1.099	0.850	1.092	1.017	0.911

Effch represents technical efficiency, techch represents technological progress, pech represents pure technical efficiency, and sech represents scale efficiency.

**Table 6:** The annual average Malmquist index values for the three provinces and one city in the Yangtze River Delta from 2015 to 2020.

Province	2015–2016	2016–2017	2017–2018	2018–2019	2019–2020
Anhui	0.974	1.044	0.845	0.957	0.629
Jiangsu	0.908	0.972	0.937	0.953	0.770
Zhejiang	0.955	0.979	0.985	0.977	0.808
Shanghai	0.974	1.014	0.915	0.992	0.873
The Yangtze River Delta	0.948	1.003	0.913	0.962	0.728

**Figure 2:**
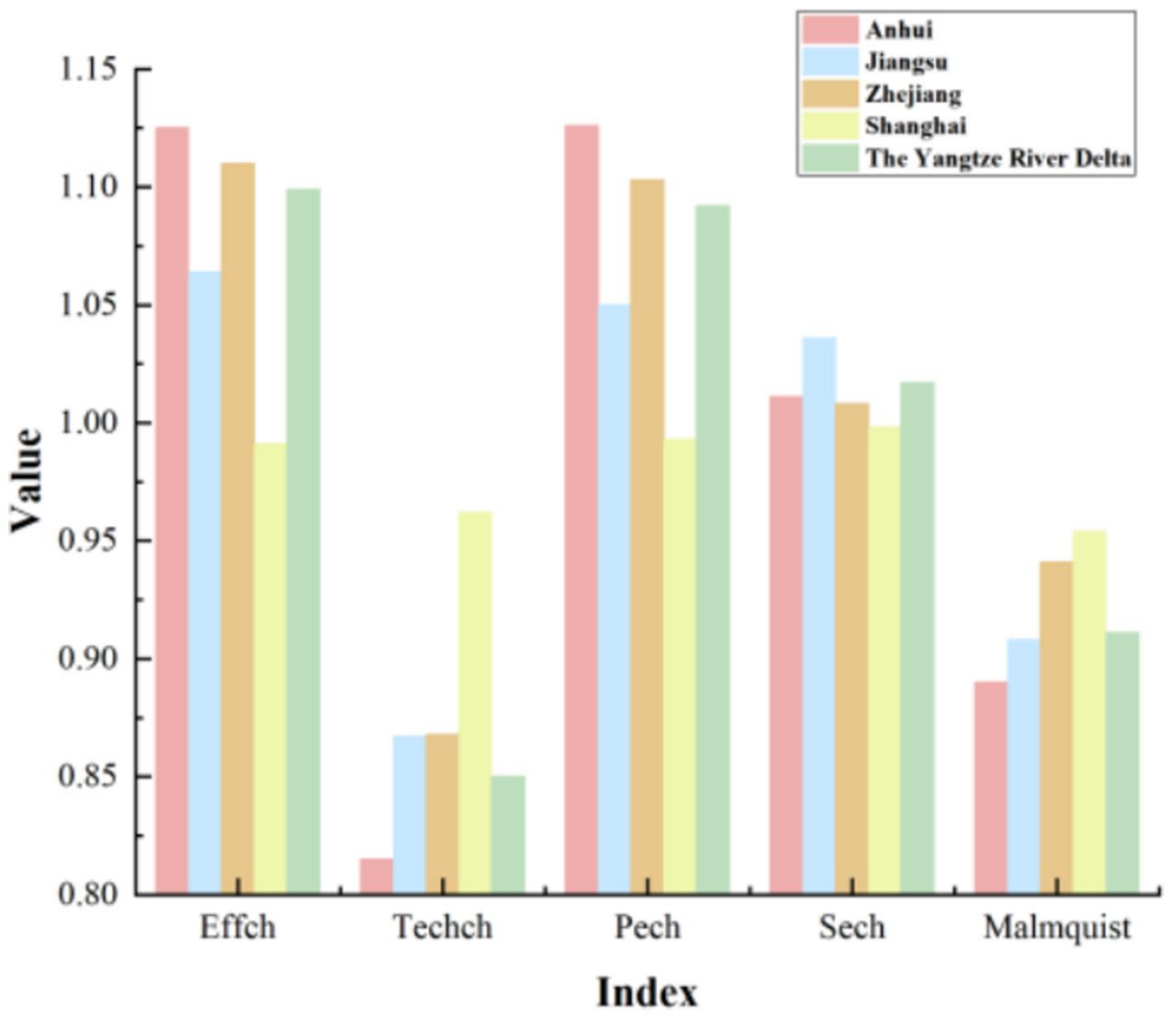
The average Malmquist decomposition efficiency in the YRD region.

**Figure 3:**
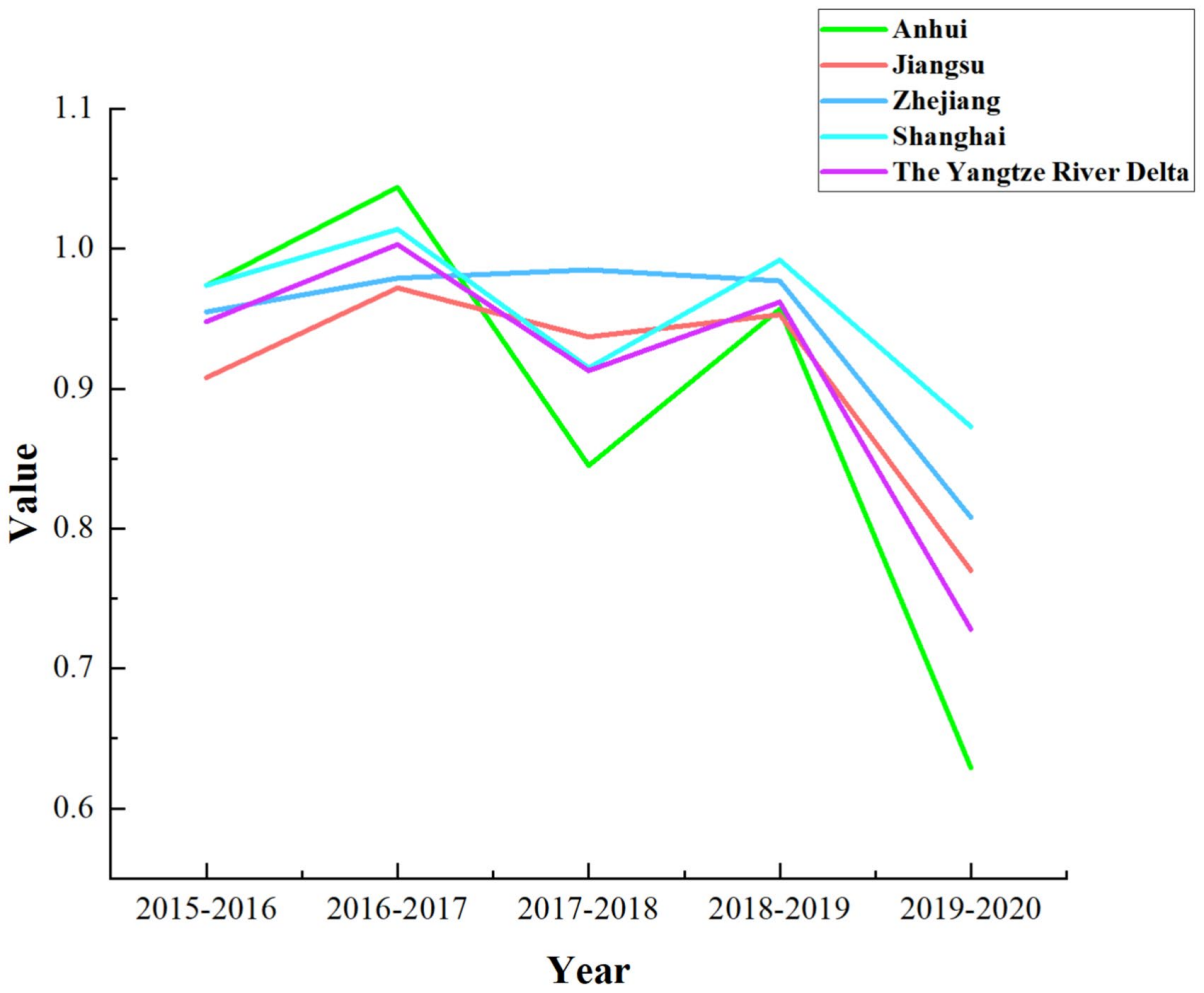
The average Malmquist index value for the YRD region from 2015 to 2020.

According to Table 5, it is evident that the overall technical efficiency, pure technical efficiency, and scale efficiency of the YRD urban agglomeration are all greater than 1. In contrast, the technological progress efficiency is less than 1, and the Malmquist index is close to 1. This indicates that during the period from 2015 to 2020, the overall medical and health resource allocation efficiency in the YRD urban agglomeration remained relatively stable but exhibited a slight decline. The primary factor constraining the comprehensive index of total factor productivity is the relatively low technological progress index, despite the relatively high scale efficiency. A more intuitive representation of this phenomenon is presented in Figure 2. As shown in Figure 2, except for Shanghai, where the decomposition efficiency values are relatively close and the technological progress index is slightly lower than other efficiency values, all provinces and the overall mean of the YRD demonstrate a significant decrease in the technological progress index compared to other indices. This situation reflects uneven resource allocation, challenges in fully leveraging resources, insufficient technological innovation and service model optimization, as well as difficulties in adapting to shifts in medical market demand within the YRD urban agglomeration. To uncover the specific factors constraining the technological progress index of the YRD, further investigation into the influencing elements is required. An analysis of the overall average Malmquist index for the three provinces and one municipality based on Table 5 reveals a ranking during the sample period as follows: Shanghai > Zhejiang > Jiangsu > Anhui. The lowest average Malmquist index in Anhui province is primarily driven by its technological progress index. Consequently, Anhui province should focus on advancing technological progress, enhancing medical innovation management capabilities, increasing the technological sophistication of medical resources, and actively introducing cutting-edge medical equipment and skilled healthcare professionals, thereby promoting the efficiency of medical and health resource allocation. Moreover, there exists a noticeable disparity in the level of medical advancement among the three provinces and one municipality, which highlights the uneven utilization of medical and health resources in the YRD region. On the other hand, this disparity also offers insights for resource allocation strategies in less developed areas. The three provinces and one municipality should further enhance mutual communication and learning, promote the inter-regional circulation of medical and health resources, and thereby continuously improve the overall efficiency of medical and health resource allocation in the YRD region. As shown in Figure 3, the dynamic efficiency values of the YRD region exhibit an “M”-shaped trend, indicating that the allocation of medical and health resources in the YRD urban agglomeration has experienced unstable development over the 6 years, influenced by both internal and external factors. Moreover, according to the statistics in Table 6, the average Malmquist index value of the YRD region exceeded 1 only in 2017, likely due to the significant progress made in deepening the reform of China’s medical and health system that year. The government implemented multiple reform measures, which contributed to the improvement of the comprehensive efficiency of medical and health resource allocation in 2017. The most substantial decline occurred in 2020, primarily due to the impact of the epidemic, which caused nationwide shortages of medical resources and led to a sharp drop in the comprehensive efficiency value of the YRD region.

Table 7 shows the changes in the Malmquist index of the average medical and health resource allocation efficiency of 41 cities in the YRD urban agglomeration from 2015 to 2020, as well as the changes in their efficiency decomposition indexes. Looking at the decomposed efficiency values in Table 7, it is evident that the technological progress indexes for all cities are less than 1, which is the primary factor constraining the Malmquist total factor indexes for most cities. Only Shanghai has a technological progress index greater than the pure technical efficiency index, indicating that for Shanghai, the constraint on its medical resource allocation efficiency is more prominently driven by changes in pure technical efficiency. Regarding pure technical efficiency, most cities have values above 1, but Tongling, Xuzhou, Bozhou, Quzhou, Huai’an, Changzhou, and Shanghai still need to further enhance their pure technical efficiency. This necessitates continuous improvement in decision-making capabilities and the management and utilization of existing technologies to promote overall efficiency gains. In terms of scale efficiency, Bozhou has the lowest scale efficiency among the cities in the YRD urban agglomeration, with a value of 0.973, which is close to 1. This indicates that the overall scale efficiency index for the cities in the YRD urban agglomeration of China has reached a relatively high level.

**Table 7:** The average Malmquist index and its decomposition of medical and health resource allocation efficiency for 41 cities in the Yangtze River Delta urban agglomeration from 2015 to 2020.

Region	Effch	Techch	Pech	Sech	Malmquist	Rank
Hefei	1.152	0.894	1.136	1.020	0.989	2
Huaibei	1.157	0.802	1.232	0.994	0.866	33
Bozhou	0.929	0.844	0.955	0.973	0.791	40
Suzhou	1.067	0.810	1.021	1.066	0.866	32
Bengbu	1.156	0.824	1.157	0.998	0.939	16
Fuyang	1.120	0.814	1.139	0.985	0.908	21
Huainan	1.040	0.789	1.056	0.987	0.822	39
Chuzhou	1.041	0.800	1.098	0.974	0.828	38
Lu’an	1.316	0.837	1.286	1.013	1.002	1
Maanshan	1.310	0.759	1.201	1.054	0.890	27
Wuhu	1.097	0.859	1.114	0.982	0.944	13
Xuancheng	1.171	0.787	1.181	0.992	0.880	30
Tongling	0.970	0.872	0.904	1.102	0.853	36
Chizhou	1.125	0.753	1.179	1.033	0.790	41
Anqing	1.193	0.800	1.193	0.994	0.966	7
Huangshan	1.163	0.799	1.162	1.002	0.902	24
Nanjing	1.031	0.902	1.032	1.014	0.924	19
Wuxi	1.032	0.899	1.042	0.990	0.922	20
Xuzhou	1.005	0.889	0.913	1.141	0.894	25
Changzhou	1.078	0.865	0.981	1.098	0.903	23
Suzhou	1.000	0.907	1.004	0.997	0.907	22
Nantong	1.080	0.830	1.052	1.029	0.886	29
Lianyungang	1.055	0.884	1.087	0.987	0.926	18
Huai’an	1.056	0.821	0.977	1.161	0.856	35
Yancheng	1.065	0.850	1.136	1.010	0.887	28
Yangzhou	1.161	0.804	1.168	1.023	0.892	26
Zhenjiang	1.111	0.905	1.109	1.002	0.981	5
Taizhou	1.075	0.834	1.077	0.997	0.876	31
Suqian	1.086	0.884	1.071	1.014	0.951	12
Hangzhou	1.042	0.909	1.043	0.998	0.942	14
Ningbo	1.110	0.861	1.101	1.009	0.937	17
Wenzhou	1.001	0.868	1.001	0.999	0.862	34
Jiaxing	1.116	0.878	1.119	1.001	0.952	10
Huzhou	1.197	0.842	1.186	1.002	0.960	8
Shaoxing	1.051	0.892	1.055	0.994	0.939	15
Jinhua	1.172	0.856	1.167	1.004	0.985	4
Quzhou	0.994	0.855	0.966	1.048	0.853	37
Zhoushan	1.241	0.879	1.217	1.019	0.979	6
Taizhou	1.143	0.842	1.134	1.007	0.951	11
Lishui	1.146	0.866	1.141	1.005	0.986	3
Shanghai	0.991	0.962	0.993	0.998	0.954	9

Effch represents technical efficiency, techch represents technological progress, pech represents pure technical efficiency, and sech represents scale efficiency.

### 3.3 Factor analysis based on the panel Tobit model

Based on the preceding analysis, it is clear that there are substantial disparities in the efficiency of medical and health resource allocation among cities within the YRD urban agglomeration. To further investigate the critical factors influencing this efficiency, this study employs the comprehensive efficiency values derived from the undesirable super-efficiency SBM model as the dependent variable. Eight key influencing factors, categorized into three dimensions—economic and social environment, fiscal support level, and internal healthcare system—are selected as independent variables. Tobit regression analysis is performed using Stata 17.0 software to evaluate the factors affecting the efficiency of medical and health resource allocation across different regions, with the results displayed in Table 8.

**Table 8:** The panel Tobit regression results of medical and health resource allocation efficiency in the Yangtze River Delta urban agglomeration from 2015 to 2020.

Variables	Regression coefficient	Standard error	z-values	*p-*values
Urbanization rate	0.132	0.303	0.44	0.664
Per capita GDP	0.022^**^	0.011	1.96	0.050
Urban–rural income gap index	0.288^***^	0.063	4.54	0.000
Proportion of healthcare fiscal expenditure	1.019	0.962	1.06	0.290
Fiscal autonomy	0.096	0.145	0.66	0.509
Doctor-to-nurse ratio	0.179	0.158	1.13	0.257
The ratio of medical and nursing staff to health technical personnel	−2.148^***^	0.659	−3.26	0.001
The average length of hospital stay for discharged patients	0.033*	0.019	1.77	0.077
_cons	0.996	0.603	1.65	0.099
Wald chi2(8)	63.14			
Prob > chi2	0.000			

***, **, and * denote significance at the 1, 5, and 10% levels, respectively.

The regression results indicate that the Wald chi2 value is 63.14 and is significant at the 1% level, indicating that there are no issues such as serial correlation or heteroscedasticity in the regression model.

Research indicates that the regression coefficient between the urbanization rate and the efficiency of medical and health resource allocation in the YRD urban agglomeration is 0.132, yet it fails to pass the significance test. While an increase in the urbanization rate theoretically implies a stronger capacity for medical and health resource allocation, which aligns with the findings of this study, the insignificant relationship might stem from the relatively robust urbanization foundation already present in the YRD urban agglomeration. This pre-existing strength diminishes the marginal impact of urbanization on resource allocation efficiency, thereby reducing the statistical significance of the results. Consequently, cities within the YRD urban agglomeration that exhibit lower urbanization rates should prioritize further urbanization efforts to enhance their capacity for medical and health resource allocation.

Per capita GDP is significantly and positively correlated with the efficiency of medical and health resource allocation in the YRD urban agglomeration. As a key indicator of regional economic development, per capita GDP not only reflects the level of economic prosperity but also determines the capacity for investment and coordinated management across various sectors. This suggests that an increase in per capita GDP typically drives regional economic growth, which in turn plays a crucial role in enhancing the efficiency of medical and health resource allocation. Consequently, variations in per capita GDP have a substantial positive impact on the efficiency of medical and health resource allocation.

The urban–rural income gap index is significantly positively correlated with the efficiency of medical and health resource allocation. There exists a notable disparity in the distribution of medical and health resources between urban and rural areas in China. Urban areas tend to have a denser concentration of these resources, whereas rural regions exhibit a clear deficit in comparison. Additionally, healthcare consumption patterns differ markedly between urban and rural populations in China, with urban residents spending considerably more on healthcare services than their rural counterparts. Consequently, a reduction in the urban–rural income gap often implies that urban income growth rates are lower than those in rural areas, potentially leading to decreased medical consumption in cities. This could result in underutilized medical resources and reduced efficiency in resource allocation. However, this trend may be specific to the sample period analyzed. For instance, during the COVID-19 pandemic, the well-developed medical system in the YRD region attracted patients from other areas for treatment. This influx led to the efficient utilization of medical and health resources and an improvement in resource allocation efficiency despite the widening income gap. Therefore, the urban–rural income gap index remains significantly positively correlated with the efficiency of medical resource allocation.

The proportion of healthcare fiscal expenditure is positively correlated with the efficiency of medical and health resource allocation. This suggests that a higher proportion of healthcare fiscal expenditure in the government’s general public budget expenditure enhances the capacity to effectively utilize medical equipment, talents, and other resources, thereby improving the efficiency of medical and health resource allocation. Therefore, the government should continue to increase fiscal investment in medical and health care within the YRD urban agglomeration to sustainably enhance the efficiency of medical and health resource allocation.

Fiscal autonomy positively influences the efficiency of medical and health resource allocation. Local governments with greater fiscal autonomy are better positioned to understand their regional financial needs, thereby enabling them to enhance the rationalization of medical and health resource distribution. Moreover, fiscal freedom empowers governments to swiftly adjust budgets in response to public health emergencies. Additionally, fiscal autonomy fosters the establishment of performance accountability mechanisms and enables local governments to improve healthcare resource utilization efficiency through technological innovations such as PPP models and digital management systems. However, the insignificant results observed may stem from the fact that the benefits of fiscal autonomy often rely on complementary systems, such as regulatory transparency and robust performance appraisal frameworks. Consequently, the gradual expansion of fiscal autonomy should be continuously promoted alongside the improvement of supporting mechanisms to fully unlock the financial potential for healthcare optimization.

The doctor-nurse ratio is positively correlated with the efficiency of medical and health resource allocation, although the correlation is not statistically significant. Practicing physicians and nurses constitute an integral part of medical resources and are also key users of other medical resources. Thus, maintaining an appropriate doctor-nurse ratio can facilitate the effective allocation of medical resources. A positive regression coefficient suggests that in the future, the YRD urban agglomeration should continue to increase the number of practicing physicians to further enhance the efficiency of medical resource allocation.

The ratio of medical and nursing staff to health technical personnel is significantly negatively correlated with the efficiency of medical and health resource allocation. Health technical personnel encompass a wide range of medical professionals, including physicians, laboratory technicians, pharmacists, and others. An increase in the number of health technical personnel can substantially enhance the management capacity, service delivery, and quality of other medical and health resources, thereby promoting the rational use of these resources. The current state of medical human resources in China reveals a shortage of highly educated health technical personnel and relatively insufficient medical research capabilities. A higher proportion of practicing physicians and registered nurses relative to health technical personnel may result in fewer pharmacists, laboratory technicians, and other technical personnel, potentially leading to an imbalance in medical and health resource allocation and reducing the efficiency of such allocation. To improve the efficiency of medical and health resource allocation in the YRD urban agglomeration, it is essential to prioritize human resource allocation, establish a reasonable balance among different types of personnel, and ensure the coordinated operation of the medical resource system.

The average length of hospital stay for discharged patients exhibits a significantly positive correlation with the efficiency of medical and health resource allocation. However, given that the variation in the average length of hospital stay across different regions is relatively small due to patient needs and treatment restrictions, the resulting correlation coefficient tends to be low. The positive correlation can be attributed to the extended average length of hospital stay for discharged patients. This extension enhances the utilization of medical resources, such as beds and equipment, by allowing them to operate more continuously. It also reduces redundancy and constraints in resource allocation, thereby improving the efficiency of medical and health resource utilization.

## 4 Discussion

### 4.1 Discussion 1: differences in the efficiency of medical and health resource allocation across different cities

There are significant variations in efficiency among the 41 cities within the YRD urban agglomeration. Cities such as Shanghai, Tongling, Suzhou, and Bozhou demonstrate relatively higher efficiency, whereas cities like Quzhou, Lishui, and Lianyungang exhibit lower efficiency levels. From an economic standpoint, Shanghai and Suzhou, as two major economic hubs in the region, benefit from rapid economic growth that enables substantial investment in medical infrastructure, technological innovation in healthcare, and the recruitment of high-caliber medical professionals. Furthermore, national policy support and more systematic resource allocation strategies have created favorable conditions for enhancing the efficiency of medical and health resource distribution. Conversely, less efficient cities such as Quzhou and Lishui lack these advantageous conditions. However, economic development is a long-term endeavor, and fostering economic progress demands sustained effort and strategic planning. Consequently, it is unrealistic to expect cities with low medical efficiency to improve solely through rapid economic growth. In this context, cities like Bozhou, which achieve high efficiency despite having an average economic level, serve as exemplary cases. The Bozhou municipal government prioritizes the development of the medical industry by formulating and continuously refining policies aimed at constructing a robust healthcare service system. Specific themes, key measures, and effectiveness evaluations of these policies are outlined in Table 9.

**Table 9:** Analysis of medical policy measures and their effectiveness in Bozhou city.

Policy themes	Main measures	Effectiveness analysis
Recruitment and training of health personnel	Implement special “college student rural doctors” program and free tuition project for oriented medical students in rural areas.	Promoting the construction of medical talent teams in Bozhou city, and even less developed cities have access to professional medical and health personnel resources.
Public health service capability	Strengthen the standardized construction of public health departments in medical and health institutions, and implement the application of regional disease surveillance, early warning, and emergency command scenarios.	Enhancing the efficiency of the public health system to safeguard people’s health and ensure the stability of the healthcare system.
Complementarity of urban and rural healthcare resources	Strengthen the lead role of county-level hospitals while consolidating the grassroots healthcare network to enhance urban–rural medical collaboration.	Enhancing the coverage and efficiency of high-quality healthcare services to promote balanced development of medical capabilities.
Medical technology innovation	Promote clinically oriented research grounded in original innovation to address cutting-edge medical technologies and health needs, and facilitate the translation and implementation of clinical research outcomes	Enhancing clinical competence, advancing healthcare industry transformation, optimizing medical services, and improving public health outcomes.
Utilization of medical resources	Guide and support public hospitals with low bed occupancy rates to transform into nursing homes and rehabilitation hospitals, increasing the number of rehabilitation and nursing institutions.	Reduce idle and redundant medical resources and improve the efficiency of medical and health resource utilization.

Through the implementation of the above policies, the efficiency of medical and health resource allocation in Bozhou City has been effectively improved and developed. Given that Bozhou’s economic level and other conditions are relatively similar to most cities with low efficiency, drawing more lessons from Bozhou’s medical-related policies can provide strong support and assistance to low-efficiency cities, playing a significant role in enhancing their medical efficiency.

### 4.2 Discussion II: the path to breakthroughs in medical technology advancement

In the analysis of medical and health resource allocation efficiency, advancements in medical technology play a pivotal role in enhancing medical efficiency and achieving optimal output with minimal input. With regard to static efficiency, the variations observed in the three provinces and one municipality of the YRD during the sample period were predominantly attributed to fluctuations in pure technical efficiency. Concerning dynamic efficiency, the technological progress indices of all cities in the YRD remained below 1, and the relatively low level of technological progress serves as the primary constraint on the total factor productivity composite index.

Under the rapid development of new-generation information technologies, the advancement of artificial intelligence (AI) technology plays a critical role in improving the efficiency of medical and health resource. Current research demonstrates that AI-driven approaches significantly enhance medical diagnosis (27), healthcare services (28), and healthcare systems (29), with AI applications proving highly effective in boosting the efficiency of medical and health resource allocation. Furthermore, the development of health information systems provides strong support for reducing clinical errors, assisting healthcare professionals, and improving the efficiency of medical care (30). Additionally, in the context of the internet era, telemedicine technology has been implemented to address the inequitable distribution of medical resources. Its adoption substantially enhances the efficiency of medical resource allocation and improves healthcare performance in low-efficiency cities (31). However, the adoption and implementation of these technologies face varying challenges across different urban environments. Existing studies indicate that health information technologies carry inherent risks, including high costs of modern information systems and potential system failures that may negatively impact both patients and healthcare staff (32).

In the YRD urban agglomeration, the utilization of technologies such as AI diagnosis, health information systems, and telemedicine differs across cities, which is an important factor contributing to efficiency disparities among them. Here, to minimize the influence of factors like economic and technological capabilities that are difficult to rapidly develop and change in the short term, we select Bozhou and Lianyungang for case comparison, analyzing the obstacles to healthcare technology application and strategies for promotion. As typical representatives of healthcare efficiency in the YRD region, Bozhou City has achieved an efficient allocation of medical and health resources through systematic technology integration, while Lianyungang City shows obvious structural imbalances in healthcare technology application. The comparison between the two is shown in Table 10.

**Table 10:** Comparative analysis of efficiency differences between Bozhou city and Lianyungang city.

Comparative dimensions	Bozhou city	Lianyungang city
Policy support	Implement special smart healthcare action plans, driven by both fiscal subsidies and performance evaluations, to systematically promote technology implementation.	Imbalance in the allocation of resources, with financial investment concentrated in the head medical institutions and a shortage of funds for the smart transformation of grass-roots units.
Infrastructure development	Advance deployment of a comprehensive 5G medical private network to ensure high-definition image transmission and teleconsultation. Iintegrate blockchain technology into medical data platforms.	Grassroots medical institutions lack basic information systems, with coexisting idle high-end equipment and gaps in fundamental technologies.
Technology integration capability	Establish an “AI + Regional Imaging Center” collaborative network to achieve data interoperability and service coordination through a tiered diagnosis and treatment system.	Low compatibility between intelligent diagnosis systems and local information systems results in difficulties in achieving seamless workflow integration.
Resource allocation efficiency	Data-driven performance management to reduce duplicate diagnoses and optimize resource allocation.	Grassroots-level technological application gaps coexist with low utilization rates of advanced equipment, resulting in prominent contradictions between resource wastage and unmet medical demands.
Talent reserves	Coordinate technological collaboration through municipal-level platforms to enhance multi-tier healthcare institution synergy.	Insufficient reserves of interdisciplinary talents (clinical + technological application) lead to diminished technology utilization efficacy.

Based on the comparative analysis of the two places, this study proposes a “four-dimensional synergy” strategy system. The first is the differentiated path of technology introduction, which promotes technology application and discovery according to local conditions. High-efficiency regions focus on the development of intelligent decision support systems and deepen the value mining of clinical data; low-efficiency regions prioritize the deployment of lightweight cloud service platforms and lower the threshold of technology use through the SaaS model. After that, we will promote the implementation of the progressive mechanism for capacity building, build a “technology hosting” support system, and promote the technological radiation of high-quality medical resources. In addition, we will learn from high-efficiency cities to formulate standards for assessing the effectiveness of medical technology applications, which will be incorporated into the performance appraisal system of medical institutions. In addition, institutional innovation safeguards have been implemented, a provincial medical technology special fund has been set up, and the cost-sharing and benefit-sharing mechanism has been improved. Promote the formation of government-industry-university-research collaborative innovation alliances to facilitate the precise docking of technology research and development with clinical needs. Finally, promote the updating of talent training mechanisms, strengthen the training of complex talents and the talent incentive mechanism.

### 4.3 Discussion III: safeguards for the long-term effectiveness of policies

Healthcare policies play an important role in improving the efficiency of medical and health resource allocation. The average dynamic efficiency of medical and health resources allocation in the YRD urban agglomeration is “M”-shaped, showing an unstable trend, which is significantly affected by changes in policies and internal and external environments. Only in 2017 was the average Malmquist index of the region greater than 1, which is attributable to the remarkable achievements made by China in deepening the reform of its medical and healthcare system in that year. 2017 saw the implementation of a comprehensive reform to improve the efficiency of medical care, health insurance and pharmaceuticals. On the health insurance front, it adjusted the health insurance catalog, promoted the convergence of national drug price negotiations and health insurance, and implemented policies such as major disease insurance. On the healthcare front, China is pushing for an increase in the scope of pilot medical reforms, the implementation of hierarchical diagnosis and treatment, and the strengthening of medical service reforms, among other measures. On the pharmaceutical side, it has implemented comprehensive monitoring and reforms from research and development, production, procurement, distribution to utilization, and multi-level measures to control pharmaceutical technology improvement and quality enhancement. Through these policy reforms, medical efficiency in the YRD urban agglomeration gained a high level of improvement in 2017. However, the subsequent medical efficiency then showed a slow decline again, excluding the impact of unconventional factors such as epidemics, the issue of long-term policy effectiveness is also a concern and problem to be solved. As China’s maritime neighbor, Japan maintains a leading level of the efficiency of medical and health resource allocation, and there are many lessons to be learned from its healthcare standards and policies. First, Japan has implemented a universal health insurance system with mandatory enrollment but a cost-sharing strategy, which not only reduces the burden of individuals but also avoids the misuse of medical resources. Secondly, Japan has implemented stringent medical price control and promoted government-led pricing and a “drug price differential system,” which has well reduced the cost of medication for residents, thereby alleviating the problem of idle medical resources brought about by economic strength. Third, Japan promotes primary care is limited, the establishment of prevention-oriented hierarchical diagnosis and treatment system, can mobilize the use of primary care resources, and improve the efficiency of use. Fourth, Japan’s policy calls for a nationally unified electronic medical record system and the implementation of comprehensive information management to achieve balanced regional resource allocation. Fifth, Japan has implemented continuous policy optimization and innovation, and promoted the establishment of a dynamic adjustment mechanism to improve the dynamics of policies according to the actual situation, to achieve the effectiveness of long-term healthcare policies. Therefore, to improve the effectiveness of long-term policies, cities in the YRD urban agglomeration should learn from international experience and promote the continuous optimization and improvement of policies, to promote the stable enhancement and progress of the efficiency of medical and health resource allocation.

### 4.4 Discussion IV: crisis resilience development

The most significant decrease in the overall dynamic efficiency of the YRD urban agglomeration is in 2020, when the impact of an epidemic led to a shortage of medical and health resources across the country, which in turn led to a sharp decrease in the overall efficiency value of the YRD urban agglomeration. This reflects the fact that the ability of the healthcare system to respond promptly is a particularly important aspect in the event of an outbreak. A resilient health system can prepare for, respond to, and adapt to disruptive public health events while ensuring continuity in the delivery of high-quality essential health services at all levels of the health system (33). Therefore, how to increase the resilience of the healthcare system to future crises is also an important element to be discussed in the findings of this study. Health systems need to rapidly reorganize their resources and clinical services in the face of a crisis to minimize the risk of healthcare-related transmission and to meet the public health requirements of continuous surveillance, risk mitigation, and containment (34).

In the face of the outbreak, China has proposed measures such as adaptive governance, a culture of ethical compliance, trust, and collaboration, and the use of artificial intelligence, cloud computing, and other technologies to rapidly monitor and manage the outbreak (35). These measures have also done a good job of controlling the outbreak and guarding people’s lives and health, but the impact of COVID-19 on the medical and health resource allocation is indeed obvious. Singapore was also one of the first countries to be affected by COVID-19, and the resilience of its health system was also greatly challenged during the spread of the epidemic. However, Singapore adapted its health system through extremely rapid response efforts, and these measures have some lessons for improving crisis resilience (36). Singapore has made efforts to improve healthcare service delivery by prioritizing the establishment of a dedicated infectious disease center for outbreak management, guaranteeing specialized healthcare service delivery, and responding quickly to emergencies (37). Structured training is used to improve the professionalism of healthcare workers and reduce their vulnerability when a health crisis strikes (38). Rapid development of the COVID-19 interim measures bill and control orders, thus providing relief from the demand for healthcare resources (36). Overall, Singapore demonstrated sufficiently strong crisis resilience during the outbreak due to its clear leadership and governance, accurate and transparent government communication, dynamic adoption of public health measures, access to crisis financing, and complementary policy-legal foundations (36). In addition, WHO, in the course of COVID-19, has emphasized the importance of incorporating digital health technologies to facilitate the stockpiling of key strategic resources and the training of government, healthcare workers, and other subjects in emergencies. In the face of future uncertainty, it is difficult to accurately predict whether another health emergency will occur. Therefore, the YRD urban agglomeration needs to learn from its own experience and that of international organizations to improve its crisis resilience through healthcare resource stockpiling and emergency management, to reduce the impact of emergencies on the efficiency of medical and health resource allocation, and to ensure that the healthcare system and resource allocation operate rationally.

### 4.5 Discussion V: the structural barriers to the urban–rural health-care gap

In the panel Tobit regression results of this study, it can be found that the urbanization rate has a positive effect on healthcare efficiency, indicating that the higher the degree of urbanization, the higher the healthcare efficiency will also be, so reducing the urban–rural gap is particularly important. However, this study also finds an extremely interesting result that the urban–rural income gap is positive for healthcare resource allocation efficiency, and although this positive relationship is very weak, the positive relationship that emerges here is well worth exploring. Increased urbanization tends to imply a more intensive allocation of medical and health resources, more synergistic and advanced infrastructure and information technology, more accessible policy implementation and quality regulation, and greater prominence of skilled and human resources. Therefore, the positive relationship between increased urbanization and medical and health resource allocation efficiency is obvious. The positive relationship between the urban–rural income gap and the allocative efficiency of medical and health resources is more elusive. The process of increasing the urban–rural income gap is often also accompanied by an increase in per capita GDP, so the increase in the urban–rural income gap does not mean that the income of urban residents increases and the income of rural residents decreases, but rather that the income of urban residents grows faster in relative terms. At the same time, in our basic data can be seen, urban residents tend to be larger than rural households in the allocation of medical expenditure, so in the process of faster growth in the income of urban residents also means that the urban medical resources are more fully utilized to improve the efficiency of medical care. At the same time, the increase in income does not mean that the medical level of progress, the huge gap between urban and rural medical levels will lead to rural residents in the medical treatment choosing a more advanced medical environment, and therefore will increase the phenomenon of rural households in the urban medical facilities for treatment, which will also increase the use of urban medical facilities, leading to the rural grass-roots health care resources idle, and in the long term is not conducive to the comprehensive efficiency of medical and health resource allocation. Therefore, how to overcome the structural barriers that impede the efficient allocation of resources to rural areas is also an issue that needs to be discussed in the findings of this study.

Existing studies have found large disparities between urban and rural areas in terms of access to health information (39), health insurance and access to care (40), medical personnel and infrastructure (41). At the same time, the distribution of medical resources between urban and rural areas still exists, such as the lack of medical investment in rural areas, the blind expansion of large hospitals in urban areas, and the concentration of advanced medical technology in urban areas (41). It is particularly important to promote rural medical incentive programs, telemedicine investment and medical infrastructure construction. In addition, this study combines the current situation of healthcare distribution in the YRD urban agglomeration and argues that four dimensions, namely, resource allocation, technological empowerment, grassroots capacity building, and policy optimization, are needed to crack the structural barriers to the effective distribution of resources to rural areas. Firstly, resource allocation should be optimized by promoting the sinking of medical factors and network layout, optimization of the pharmaceutical supply chain and balanced infrastructure. Secondly, technological empowerment should be promoted to bridge the urban–rural healthcare gap with digitalization, and to achieve accurate intervention of big data and full coverage of telemedicine. Thirdly, we should promote grass-roots capacity building, and activate the endogenous power of rural medical care through community participation and innovation of the talent “attraction, training and retention” mechanism. Finally, we should optimize the policy, and build a fair-oriented medical system framework through the tilting of medical insurance policy to the rural areas, and the reform of the financial compensation mechanism. Of course, the most important thing is to continuously monitor and improve the system and to adjust and improve the measures in the light of the actual situation, to gradually break the structural obstacles of the urban–rural healthcare gap.

### 4.6 Discussion VI: inefficient access to urban finance

Government financial support is an important source of access to funds for healthcare construction, and government investment in healthcare infrastructure in underdeveloped regions is considered an important strategy to minimize healthcare inequality (42). In the analysis of the empirical results of this study, healthcare financial expenditure positively affects the efficiency of medical and health resource allocation. The positive relationship of healthcare financial expenditure represents a higher share of healthcare financial expenditure, and more adequate healthcare funding, which in turn also tends to imply more healthcare resources equipped and updated, and more efficient healthcare. Government investment in healthcare in inefficient areas should be a key strategy to achieve equity in the distribution of different regions (43). In inefficient cities, there are problems such as dependence on a single source of finance, lag in policy implementation, and crude distribution patterns in the acquisition and pre-distribution of healthcare funds. This study suggests that the financial challenges of inefficient cities can be mitigated by constructing an “Efficiency-oriented, Multifaceted, and Dynamically-adapted” financial allocation system. First, we can learn from the Karnataka PPP model to reduce operating costs by outsourcing dialysis services, promote public-private partnerships (PPPs), and promote risk-sharing mechanisms and service outsourcing innovations to alleviate the pressure on the government while releasing financial resources for key technology upgrades. Secondly, we can implement the transformation of the results-based funding (RBF) model, promote the binding of performance indicators and dynamic adjustment algorithms, etc., and utilize big data to build a funding allocation model, to improve the efficiency of funding allocation. Such a model has been implemented to some extent in Mexico and Hangzhou, and some results have been obtained. Thirdly, it can promote system optimization and collaborative governance through the reconstruction of the regulatory framework and the construction of cross-regional collaboration platforms, to regulate the transparency of the flow of government funds while driving the participation of other stakeholders, reduce the duplication of costs of medical and health resources, and improve the efficiency of funds in inefficient cities. For example, the cross-state collaboration of Cleveland Medical Center is a successful case of cross-regional collaboration platform construction. In conclusion, to promote the reform of inefficient healthcare funding allocation, it is necessary to replace “incremental dependence” with “innovation-driven,” activate the market vitality through the PPP model, enhance the effectiveness of funding through the RBF mechanism, and strengthen the local resilience through fiscal decentralization.

## 5 Conclusion

In this study, we uncover the uneven spatial distribution and unstable temporal evolution of medical and health resources in the YRD urban agglomeration through static and dynamic efficiency analyses. We further identify underlying contradictions and issues via decomposition efficiency and Tobit regression analysis. Drawing on existing scholarly research and successful domestic and international cases, we propose recommendations for the future development and standardization of healthcare systems in the YRD urban agglomeration. These include promoting healthcare technological innovations, enhancing the long-term effectiveness of policies, strengthening resilience to crises, and dismantling structural barriers to the equitable allocation of urban and rural healthcare resources. Our findings and conclusions aim to provide evidence-based insights not only for the YRD urban agglomeration but also for other countries and cities grappling with similar challenges in medical and health resource allocation. Nevertheless, this study has certain limitations that warrant attention from future scholars. First, while the study highlights the importance of local decision-makers and mentions the promotion of collaborative governance models, it falls short in exploring the dynamics of stakeholder cooperation and participatory decision-making processes. Future research should develop a more concrete framework for collaborative stakeholder governance models. Second, although the study identifies the comprehensive influence of economic and social factors on efficiency, data collection constraints in the YRD urban agglomeration precluded the inclusion of variables such as education, housing, and employment in the analytical framework. Finally, this study conducts a comprehensive analysis of long-term effective policy reforms by integrating case comparison studies. However, it lacks sufficient incorporation of quantitative tools, such as cost–benefit analysis, to deeply explore the incremental efficiency generated by each policy. This calls for collaborative efforts from future researchers to investigate the construction of more efficient medical and health resource allocation mechanisms.

## Data Availability

The original contributions presented in the study are included in the article/supplementary material, further inquiries can be directed to the corresponding author.
